# Assessing concordance of genetic parameters between trophectoderm and inner cell mass in morphologically scored day-5 embryos via NGS

**DOI:** 10.1530/RAF-25-0157

**Published:** 2026-06-29

**Authors:** Nicole Salameh, Athina Theodosiou, Ludmila Kousoulidou, Ioannis Papaevripidou, Paola Evangelidou, Kyriaki Michailidou, Damianos Michaelides, Chloe Zoppou, Anna Agathangelou, Sozos Fasouliotis, Joanne Traeger-Synodinos, Periklis Makrythanasis, Carolina Sismani

**Affiliations:** ^1^ Cytogenetics and Genomics Department, The Cyprus Institute of Neurology and Genetics, Nicosia, Cyprus; ^2^ Biostatistics Unit, The Cyprus Institute of Neurology and Genetics, Nicosia, Cyprus; ^3^ ISIS Clinic, Nicosia, Cyprus; ^4^ Laboratory of Medical Genetics (Choremeio), Medical School, National and Kapodistrian University of Athens, Athens, Greece

**Keywords:** inner cell mass (ICM), trophectoderm (TE), mitochondrial DNA (mtDNA), preimplantation genetic testing (PGT)

## Abstract

**Abstract:**

Preimplantation genetic testing for aneuploidy (PGT-A) is used for embryo selection, on a 5–10 cell biopsy from the trophectoderm (TE), which later forms the placenta. Whether this biopsy reflects the inner cell mass (ICM), which develops into the fetus, is still a subject of debate. Various parameters were previously evaluated, many of which have been proposed as potential biomarkers based on their observed associations, including the mitochondrial DNA (mtDNA) copy number. Here, we analysed thirty-two donated blastocysts, dissected into TE and ICM, with low-pass whole-genome sequencing. For 14 of these 32 embryos, PGT-A on TE biopsy was previously performed. Ploidy status, mtDNA copy number and the morphology of TE and ICM were evaluated. 81% of blastocysts showed full ploidy concordance, and 19% showed partial concordance between their ICM and TE. Out of the 14 embryos with previous PGT-A results, 86% showed full concordance, and 14% showed partial concordance. Non-euploid blastocysts showed a significantly higher mtDNA copy number in both ICM and TE. Interestingly, among euploid blastocysts, the higher rates of mtDNA were observed in those with the best morphology of the TE alone. These results suggest that TE ploidy correctly reflects ICM, and a TE biopsy is suitable for embryo status prediction. A high mtDNA copy number in TE or ICM alone may also be indicative of aneuploidy, but a euploid embryo with a high mtDNA copy number could be suggestive of a better implantation potential. Our data support the clinical utility of PGT-A, especially when combined with additional biomarkers.

**Lay summary:**

Genetic testing before implantation is a technique used after *in vitro* fertilisation to check whether an embryo has the correct number of chromosomes, by testing a few cells from the outside layer that will form the placenta. One common concern is whether these cells accurately represent the actual cells that will develop into the baby. Here, we compared these two cell groups and observed a high consistency. We also investigated the amount of DNA copies in the cell’s energy-producing parts. Embryos with abnormal chromosome number were found to have a higher number of DNA copies than embryos with normal chromosome number. Among embryos in the latter group, those with the healthiest external appearance had the highest number of DNA copies in these energy-producing regions. These findings suggest that genetic testing before implantation is helpful and that choosing the best embryos could be further improved by considering additional biological factors.

## Introduction

Infertility is defined, by the International Glossary on Infertility and Fertility Care consensus (Zegers-Hochschild *et al.* 2017), as ‘the failure to establish a clinical pregnancy after 12 months of regular, unprotected sexual intercourse or due to an impairment of a person’s capacity to reproduce either as an individual or with his/her partner’. An increasing number of couples facing some forms of infertility turn to assisted reproductive technology (ART) procedures, such as *in vitro* fertilisation (IVF). Despite the rapid developments in ARTs, the average implantation failure is still high and is estimated at 32–51% (Harton *et al.* 2013, Cimadomo *et al.* 2021). Although there are several factors affecting IVF success, the parameters that affect embryo viability have become a major focus of investigation.

A human embryo normally reaches the blastocyst stage at day 5, forming the trophectoderm (TE) and the inner cell mass (ICM). TE gives rise to tissues of the placenta, and the ICM generates the fetus. Aiming for the highest effectiveness of the implantation process, preimplantation genetic testing (PGT) has become an integral part of assisted reproduction. More specifically, preimplantation genetic testing for aneuploidy (PGT-A) identifies aneuploidies in IVF embryos before their implantation. This screening test is performed on a 5–10 cell TE biopsy from the day-5 embryo. This enables the selective transfer of euploid embryos, aiming to reduce the missed abortions or termination of pregnancies due to embryonic aneuploidy. Even though PGT was first introduced more than 30 years ago (Handyside *et al.* 1990), it is still a controversial issue due to ethical concerns and discussions on the invasiveness in human embryos and the effect of PGT-A on the cumulative birth rates achieved through IVF (Mochizuki & Gleicher 2020).

Aneuploidy is known to be the primary cause of first-trimester miscarriages (Hassold & Jacobs 1984, Robinson *et al.* 2001), and consequently, it is one of the main factors that affect the success rate of IVF (Greco *et al.* 2014). Further to pure aneuploidies, mainly derived from meiotic oocyte errors affecting the whole embryo, mitotic errors occur during the post-zygotic stage and give rise to mosaic aneuploidies. Depending on the stage of development at which a mitotic error occurs, the level of mosaicism differs, and depending on the chromosomes involved, it determines the embryo’s fate. While meiotic errors during gamete development are mainly associated with advanced maternal age (Demko *et al.* 2016), the mitotic errors (mosaicism) identified during PGT in the early-stage embryos can be due to the technical procedure effect (Munné *et al.* 2016) or even maternal age (Cascales *et al.* 2023).

Blastocysts that undergo PGT-A can be classified as euploid, aneuploid and mosaic. Euploid embryos should be considered for transfer; however, it is now acceptable that if no euploid embryos are available, low-range (<50%) mosaic embryos should be considered, since there is no clear evidence that low-range mosaic PGT results negatively affect the outcome of implantation and pregnancy (Capalbo *et al.* 2021). The use of the low-level mosaic embryos should be prioritised by the embryo morphology (De Rycke *et al.* 2022). In addition, it is yet unclear if the chromosome involved in the mosaicism interferes with the implantation outcome (Practice Committee and Genetic Counseling Professional Group GCPG of the American Society for Reproductive Medicine 2020).

It has been shown that the human embryo exhibits a high incidence of aneuploid cells in its very early stages (Griffin *et al.* 2023), which tend to ‘normalise’ during the development (Brezina *et al.* 2011, Coticchio *et al.* 2021, Harzif *et al.* 2024). Some studies support that the ‘normalisation’ occurs between the cleavage stage (day 3) and the blastocyst stage (day 5) (Mertzanidou *et al.* 2013, Lagalla *et al.* 2017). As PGT-A at the cleavage stage involves removing only one or two blastomeres from the embryo, it can be assumed that mosaicism is identified more frequently in the blastocyst-stage biopsy, which involves 5–10 cells. Indeed, the mosaic aneuploidy detection rate with PGT-A on day 5 is relatively high (McCoy 2017, Starostik *et al.* 2020), supporting the concern for the validity and the impact of mosaic PGT-A (Gleicher *et al.* 2021) results on IVF success, since mosaic results may portend the discard of embryos, potentially leading to repetitive IVF cycles.

In addition to aneuploidy, other embryo characteristics have also been proposed as potential viability markers. The embryonic mitochondrial DNA (mtDNA) copy number has been investigated as a factor associated with embryonic development, suggesting that elevated mtDNA copy number is correlated with the blastocyst implantation failure rate (Ravichandran *et al.* 2017). The mitochondria play a crucial role in cell energy production, which in turn impacts early development.

It has been demonstrated that mtDNA replication is restricted to the early cleavage stages, resulting in a dilution of mtDNA copies in each cell division (Pikó & Taylor 1987, St John *et al.* 2010). In contrast, the replication of the mtDNA is significantly upregulated while transitioning to the blastocyst stage (Thundathil *et al.* 2005, St John *et al.* 2010). Studies in mammalian species showed that this upregulation in the blastocyst stage is restricted to the cells of the TE as they start their differentiation process in preparation for implantation. These cells are no longer pluripotent in comparison with the ICM cells, which still express pluripotency genes and are associated with low levels of expression mtDNA replication factors. A correlation between the number of mtDNA copies and the aneuploidy status of the embryo is under investigation, with some studies supporting that an elevated copy number of mtDNA is associated with a high probability of aneuploidy and hence defining the blastocyst’s future (Lee *et al.* 2019, Luo *et al.* 2023).

Previous studies, using array-based comparative genomic hybridisation (array-CGH), demonstrated high concordance of euploid, aneuploid and mosaic profiles between separated ICM and TE (Fragouli *et al.* 2008, Johnson *et al.* 2010). The recent use of next-generation sequencing (NGS) shows higher sensitivity than array-CGH, making it more suitable for the detection of mosaic aneuploidies. Hence, it is appropriate to re-evaluate the blastocyst potential, using a more sensitive method to compare ploidy concordance between ICM and TE (Friedenthal *et al.* 2018).

In response to the ongoing concern within the scientific community, we aim to investigate the hypothesis that ploidy status detected in a single 5–10 cell TE biopsy may not be present in the ICM; therefore, standard PGT-A may not accurately reflect the chromosomal profile of the whole embryo, offering limited predictive value for embryo viability. Moreover, additional biomarkers need to be considered in the overall assessment of embryo fate.

To address the above issue, we dissected 32 donated blastocyst-stage embryos into TE and ICM and applied low-pass whole-genome sequencing (LP-WGS) to identify their chromosomal profile and assess the concordance between the results obtained from the whole TE and the whole ICM of the same embryo. Moreover, for 14 of the tested embryos, PGT-A was previously performed, and the results from a single TE biopsy were available for comparison. Furthermore, the above samples were also evaluated regarding the relative mtDNA copy number.

Finally, the chromosomal profile and the mtDNA copy number were assessed for potential association with the blastocyst morphology. By examining the factors influencing embryo implantation and viability, we aimed to study the potential indicators that could help improve IVF outcomes.

## Materials and methods

This study was approved by the Cyprus National Bioethics Committee (Protocol No. EEBK/EΠ/2024/30).

### Samples

The sample group consisted of 32 cryopreserved blastocysts that had been stored for potential future use but were designated for disposal following the completion of patients’ IVF treatment. Fourteen of these had undergone PGT-A, with euploid or aneuploid results, while the remaining blastocysts had no available ploidy information before the study. No ploidy criterion was applied to sample selection, as both sub-groups included euploid and aneuploid blastocysts. Because the blastocysts were chosen solely based on the availability for disposal and not on biological characteristics, this cohort is considered to be randomly selected from a larger population of stored blastocysts. These were derived from couples who wished to terminate any ART, regardless of their IVF treatment outcome. These were single vitrification blastocysts of an average-to-excellent quality, which were thawed once for this project. Informed consent from each participant couple was obtained.

ICM and TE separation of each blastocyst was performed at a single private clinic using the RI Saturn 5™ Laser, CooperSurgical, Inc. TE-ICM dissection and isolation were performed on day 5/6 blastocysts, and samples were collected in 0.2 mL sterile tubes, containing 2 μL of 1×PBS. To minimise the risk of contamination, both the micropipetting and laser dissection steps were performed with a high degree of precision. The ICM was isolated first, followed by TE sampling. This sequence was chosen because the removal of the micropipette after ICM extraction results in a blastocyst collapse; thus, collecting the ICM before TE removal allowed for secure isolation of the ICM and minimised any chance for cross-transfer of cells.

All blastocysts were scored based on their morphology using the Gardner and Schoolcraft classification system (Gardner & Schoolcraft 1999) by specialised embryologists. The grading score includes a numerical digit between 1 and 6, indicating the degree of the expansion and the hatching status, followed by a letter between A and C, indicating the grade of the ICM, and finally, a letter between A and C, indicating the grade of the trophectoderm (Table 1). The morphology of all 32 blastocysts was of average, good, and excellent quality.

**Table 1:** The Gardner and Schoolcraft classification system.

Category/code	Description
Expansion and hatching status	
3	Full blastocyst with blastocoel filling the embryo
4	Expanded blastocyst with a blastocoel volume larger than that of the early embryo, with a thinning zona
5	Hatching blastocyst with the trophectoderm starting to herniate through the zona
6	Hatched blastocyst, blastocyst has completely escaped from zona
ICM grade	
A	Tightly packed with many cells
B	Loosely grouped with several cells
TE grade	
A	Many cells forming a cohesive epithelium
B	Few cells forming a loose epithelium

Blastocysts were derived from six couples from a total of eight IVF cycles: four couples with a maternal age between 40 and 46 years and two couples with egg donors (age≤32). From each cycle, a minimum of two and a maximum of six blastocysts were obtained. The total number of samples processed was 32 ICM and 32 TE from each of the blastocysts. Fourteen of the 32 blastocysts had previously undergone routine PGT-A testing.

### Low-pass whole-genome sequencing

Each sample was analysed by low-pass whole-genome sequencing to detect aneuploidies, including mosaic and segmental aneuploidies. LP-WGS was performed using the Embgenix PGT-A Core kit (CE-IVD) (Takara Bio Inc., USA). Amplified DNA libraries were purified and then quantified using the Qubit^TM^ dsDNA High Sensitivity Assay Kit (Thermo Fisher Scientific Inc., USA), and the pooled library was run on a MiSeq platform (Illumina Inc., USA). Analysis was performed using the Embgenix Analysis Software (RUO) (Takara Bio Inc.).

### mtDNA copy number

BAM files, which are used for storing a large amount of sequenced alignment data in NGS experiments, from all biopsies were used to estimate the relative mitochondrial DNA copy number. Initially, this approach was validated by relative mtDNA copy number calculation on 60 blind samples that had previously undergone PGT-A. All the repeated copy numbers showed a high level of uniformity, ensuring the reliability of this process. The fastMitoCalc (Qian *et al.* 2017) tool was used, and the calculations were based on the proportion of the mtDNA average coverage to the autosomal DNA average coverage. For the autosomal DNA average coverage estimation, chromosomes were randomly selected.

### Statistical analysis

PGT-A results of the ICM and TE of each blastocyst were compared to assess their concordance. When previously performed PGT-A results were available, these were included to evaluate the results of single TE biopsy. The euploid or aneuploid outcome was then assessed in relation to the corresponding mtDNA relative copy number and the blastocyst morphology.

For comparisons of the mtDNA copy number between two groups (euploid vs non-euploid), statistical differences were evaluated using a two-sample t-test, assuming equal variances. Before applying the *t*-test, the normality of residuals was assessed using the Shapiro–Wilk test to confirm that model assumptions were met. For comparisons involving more than two blastocyst morphology categories, differences in the mtDNA copy number were examined using a one-way analysis of variance (ANOVA). Post hoc pairwise comparisons were explored using two-sample Welch’s *t*-tests with Holm adjustment for multiple comparisons. In addition, the mean differences and 95% confidence intervals for the mtDNA copy number between groups were estimated. Boxplots were used to visualise how the mtDNA copy number was distributed within each group. *P*-values for each comparison are shown from the pairwise tests. All statistical analyses were performed in R version 4.4.2.

Receiver operating characteristic (ROC) curve analysis was performed to explore mtDNA copy number thresholds capable of discriminating between ploidy status and morphological grading categories. Optimal cutoffs were determined using the Youden’s index, which identifies the cutoff that maximises the combined sensitivity and specificity. The area under the curve (AUC), along with the sensitivity and specificity, is reported.

## Results

### Comparison between ICM and TE chromosomal profiles

**Figure 1:**
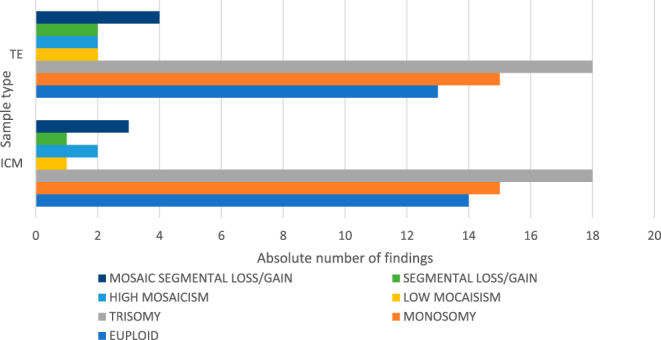
Total ploidy findings of each sample group after NGS analysis.

NGS analysis revealed 13 euploidies among the TE samples and 14 euploidies among the ICM samples. For the remaining 19 and 18 samples, respectively, a range of different findings were observed, including pure aneuploidies and mosaic aneuploidies, segmental aneuploidies and mosaic segmental aneuploidies (Fig. 1) either as single findings or as combined. Table 2 shows in detail the non-euploid findings of each ICM and TE. The results derived from the ICM and the TE of each embryo suggest that any cross-contamination during dissection and sampling was highly unlikely and would not impact the validity of PGT-A.

**Table 2:** ICM and TE NGS non-euploid findings for each embryo.

Embryo no.	Morphology score	Maternal age (years)	ICM	TE
3	5BA	<32	Euploid	Mosaic monosomy 19 (33%)
				Segmental gain 7q33q36.3 (25 Mb)
4	5BA	<32	Mosaic segmental loss 4q22.2 (97 Mb) (40%)	Mosaic segmental loss 4q22.2 (97 Mb) (26%)
5	5BB	<32	Mosaic monosomy 1 (54%)	Mosaic monosomy 1p (49%); monosomy 1q
6	5BB	<32	Segmental loss 12q22q24.33 (39 Mb)	Segmental loss 12q22q24.33 (39 Mb)
10	3BB	40–46	Monosomy 21	Monosomy 21
11	3BB	40–46	Trisomy 15	Trisomy 15
12	5BA	40–46	Trisomy 22	Trisomy 22
13	5BA	40–46	Trisomy 15	Trisomy 15
14	5BA	40–46	Monosomy 16	Monosomy 16
15	5BA	40–46	Monosomy 22	Monosomy 22
16	5AA	40–46	Trisomy 9; trisomy 11; trisomy 13; mosaic trisomy 19 (68%)	Trisomy 9; trisomy 11; trisomy 13; mosaic trisomy 19 (70%)
17	5AA	40–46	Monosomy 14; trisomy 20	Monosomy 14; trisomy 20
18	5AA	40–46	Trisomy 3; trisomy 10; monosomy 14	Trisomy 3; trisomy 10; monosomy 14
19	5AA	40–46	Trisomy 5; trisomy 11; monosomy 15	Trisomy 5; trisomy 11; monosomy 15
20	6BA	40–46	Trisomy 8; monosomy 15; trisomy 20; trisomy 21	Trisomy 8; monosomy 15; trisomy 20; trisomy 21
21	6BA	40–46	Monosomy 8; trisomy 9; monosomy 10; trisomy 11; trisomy 16; mosaic trisomy 2 (46%)	Monosomy 8; trisomy 9; monosomy 10; trisomy 11; trisomy 16; mosaic trisomy 19 (52%)
22	4BB	40–46	Monosomy 2; monosomy 7; monosomy 15; monosomy 17; trisomy 22	Monosomy 2; monosomy 7; monosomy 15; monosomy 17; trisomy 22; mosaic segmental gain 7p22.3p21.1 (19 Mb) (46%); mosaic trisomy 11 (40%)
29	5BB	40–46	Monosomy 16; mosaic segmental loss 20p (27%); mosaic segmental loss 20q (80%)	Monosomy 16
30	5BB	40–46	Monosomy 16	Monosomy 16

#### Full concordance

Twenty-six out of the 32 blastocysts showed full concordance between their ICM and TE, 13 of them having euploid concordance, seven having a single aneuploid concordance, five showing more than one aneuploidy, and finally one showing in both ICM and TE a segmental 39 Mb loss on chromosome 12 (12q22q24.33).

#### Partial concordance

Six out of the 32 blastocysts showed partial concordance between their ICM and TE, representing in all of the cases mosaic findings either of a whole chromosome or of a fragment. Embryo 3 was the only case where ICM was euploid, but TE showed a mosaic (33%) monosomy of chromosome 19 and a full segmental 25 Mb gain on chromosome 7 (7q33q36.3). One case (embryo 4) had a mosaic segmental 97 Mb loss of chromosome 4 (4q22.2), seen as a 26% mosaicism in TE and 40% in ICM. Embryo 5 had a mosaic (54%) monosomy of chromosome 1 in ICM and full monosomy of the whole long arm of chromosome 1 along with mosaic (49%) monosomy for the short arm of chromosome 1 in TE. In embryos 21 and 22, even though both had the same multiple aneuploidies in their ICM and TE, the first included a mosaic (46%) trisomy 2 in ICM and mosaic (52%) trisomy 19 in TE, and the second had additional findings in TE, a mosaic (40%) trisomy 11 and a mosaic (46%) segmental 19 Mb gain on chromosome 7 (7p22.3p21.1). Embryo 29 showed a monosomy 16 in both ICM and TE, while ICM analysis revealed an additional mosaic (27%) whole short arm loss of chromosome 20 and a mosaic (80%) whole long arm loss of the same chromosome.

### Comparison between ICM/TE and PGT-A chromosomal profiles

Available PGT-A results for fourteen of the tested blastocysts were compared with their ICM and TE results (Table 3). The results showed a high rate of concordance between previous TE biopsy PGT-A, whole ICM and whole TE results. Two embryos (embryos 8 and 9) showed an euploid profile, six (embryos 10–15) showed a single aneuploidy and six (embryos 16–20) showed the same multiple aneuploidies. However, in embryo 21, further to the identical multiple aneuploidies detected, the initial PGT-A did not show any evidence of mosaic trisomy of chromosome 2 (46%) or chromosome 19 (50%), which were detected in total ICM and total TE, respectively.

**Table 3:** ICM and TE findings of the embryos with a previous PGT-A result. Comparison between the three findings.

Embryo no.	ICM	TE	PGT-A
8	Euploid	Euploid	Euploid
9	Euploid	Euploid	Euploid
10	Monosomy 21	Monosomy 21	Monosomy 21
11	Trisomy 15	Trisomy 15	Trisomy 15
12	Trisomy 22	Trisomy 22	Trisomy 22
13	Trisomy 15	Trisomy 15	Trisomy 15
14	Monosomy 16	Monosomy 16	Monosomy 16
15	Monosomy 22	Monosomy 22	Monosomy 22
16	Trisomy 9; trisomy 11; trisomy 13; mosaic trisomy 19 (68%)	Trisomy 9; trisomy 11; trisomy 13; mosaic trisomy 19 (70%)	Trisomy 9; trisomy 11; trisomy 13; trisomy 19
17	Monosomy 14; trisomy 20	Monosomy 14; trisomy 20	Monosomy 14; trisomy 20
18	Trisomy 3; trisomy 10; monosomy 14	Trisomy 3; trisomy 10; monosomy 14	Trisomy 3; trisomy 10; monosomy 14
19	Trisomy 5; trisomy 11; monosomy 15	Trisomy 5; trisomy 11; monosomy 15	Trisomy 5; trisomy 11; monosomy 15
20	Trisomy 8; monosomy 15; trisomy 20; trisomy 21	Trisomy 8; monosomy 15; trisomy 20; trisomy 21	Trisomy 8; monosomy 15; trisomy 20; trisomy 21
21	Monosomy 8; trisomy 9; monosomy 10; trisomy 11; trisomy 16; mosaic trisomy 2 (46%)	Monosomy 8; trisomy 9; monosomy 10; trisomy 11; trisomy 16; mosaic trisomy 19 (52%)	Monosomy 8; trisomy 9; monosomy 10; trisomy 11; trisomy 16

### Comparison between mtDNA copy number and ICM/TE chromosomal profiles

To enrich the arsenal of embryo quality prediction factors, the mtDNA copy number of each ICM and TE was studied to identify any association with the chromosomal status of the blastocyst. For statistical analysis, the chromosomal status of both ICM and TE was converted into two categories: euploid and non-euploid. In general, the mtDNA copy number of each TE was significantly increased compared with the ICM of the same embryo, regardless of the chromosomal profile.

**Figure 2:**
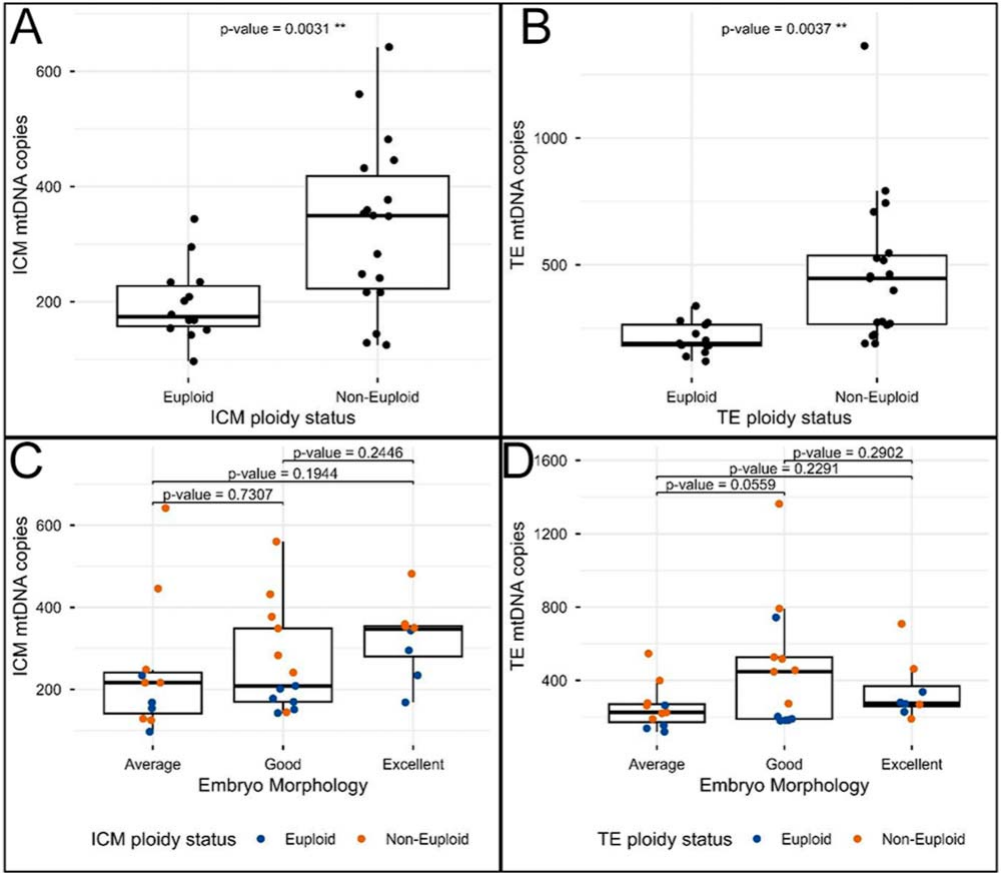
(A) Distribution of the ICM mtDNA copy numbers in the euploid and non-euploid groups. Based on the median value of each category, the non-euploid samples have higher mtDNA copy number in comparison with the euploid (** = *P* < 0.01). The whiskers of each box, excluding potential outliers, also show a larger spread in the non-euploid samples, meaning a higher variability in mtDNA copy numbers compared with the ones of the euploid group. (B) Distribution of the TE mtDNA copy number in the same two groups. Again, the non-euploid group of the TE shows a noticeably higher median mtDNA copy number value (** = *P* < 0.01), and the large box and its whiskers demonstrate a higher variability in the mtDNA copy number similar to the ICM. The association of embryo quality and the mtDNA copy number of the ICM (C) and the TE (D) was also investigated. The average, good and excellent quality groups did not exhibit any association with the mtDNA copies of either the ICM or the TE.

Figure 2A shows the distribution of the ICM mtDNA copy numbers in these two categories. Based on the median value of each category, the non-euploid samples have higher ICM mtDNA copy numbers in comparison with the euploid. The whiskers of each box, excluding potential outliers, also show a larger spread in the non-euploid samples, meaning a higher variability in mtDNA copy numbers compared with the ones of the euploid group. Some values lie outside the whiskers, indicating outlying behaviour. Performing a pairwise comparison to assess the difference between the two categories with a confidence interval of 95%, we saw a difference of 135 copies between the two, with the euploid mtDNA copy number being the low and the non-euploid being the high. This comparison can be translated to a *P*-value of 0.0031, indicating a difference of statistical significance.

Again, the non-euploid group of the TE shows a noticeably higher median mtDNA copy number value (Fig. 2B), and the large box and its whiskers demonstrate a higher variability in the mtDNA copy number similar to the ICM. Performing a pairwise comparison in TE, to assess the difference between the two categories with a confidence interval of 95%, we saw a difference of 256 copies between the two, with the euploid mtDNA copy number being the low and the non-euploid being the high. This comparison can be translated to a *P*-value of 0.0037, indicating a difference of statistical significance. An mtDNA copy number threshold of approximately 369 copies was identified, yielding a sensitivity of 58% and a specificity of 100%, indicating that mtDNA copy numbers higher than 369 in the TE were associated with non-euploid status.

To summarise, non-euploid samples tend to have a higher and more variable mtDNA copy number in both ICM and TE cell groups.

### Comparison between mtDNA copy number and morphology

The relation of embryo quality and the mtDNA copy number was also investigated. The average, good and excellent quality groups did not exhibit any association with the mtDNA copies of either the ICM or the TE (Fig. 2C and D). However, a significant difference in means of the mtDNA copy number was detected between the TE of A-score and the TE of B-score, with the former showing significantly higher values (Table 4A). No difference was observed in ICM. Overall, the mtDNA copy number of each TE was significantly elevated compared with the ICMs, irrespective of the chromosomal profile. Further analysis of the A-score and the B-score was performed for the following subcategories: euploid ICM, non-euploid ICM, euploid TE and non-euploid TE. Interestingly, it showed a statistically significant difference in comparing the two grading scores of the euploid TE group (*P* = 0.04) (Table 4B). An mtDNA copy number threshold of approximately 182 copies was identified, yielding a sensitivity of 57% and a specificity of 100%, indicating that mtDNA copy numbers higher than 182 in euploid TE were associated with A-grade TE morphology.

**Table 4:** *P*-value between the mtDNA copy number of the A-score and the mtDNA copy number of the B-score in all ICM and TE samples (A) and after further subcategorisation in euploid and non-euploid (B). The *P*-value was calculated based on the shown mean values.

	ICM			TE		
	EP + NEP	EP	NEP	EP + NEP	EP	NEP
A: all samples						
*P*-value between A-score and B-score	0.66			0.009		
Mean of mtDNA copy number of A-score	284.97			457.72		
Mean of mtDNA copy number of B-score	264.98			241.20		
B: further categorised samples						
*P*-value between A-score and B-score		0.07	0.21		0.04	0.61
Mean of mtDNA copy number of A-score		227.24	386.00		242.01	407.91
Mean of mtDNA copy number of B-score		165.02	314.96		174.35	482.93

EP, euploid; NEP, non-euploid.

## Discussion

In this study, we compared the whole ICM and the whole TE chromosomal profiles of the same embryo using NGS, investigating whether a routine single TE biopsy for PGT-A is representative of the whole embryo and thus can accurately determine its potential. NGS analysis showed a diverse range of chromosomal profiles in both ICM and TE groups, including euploid, aneuploid, mosaic aneuploid and segmental aneuploid, either mosaic or not.

According to our current results, an 81.25% concordance was found between all ICM and the corresponding TE, including euploid or full aneuploid concordance. The remaining 18.75% showed partial concordance limited to the level of mosaicism of the aberration. Most of these deviations were below the reportable cutoff level of mosaicism, based on our laboratory’s cutoff criteria, which were formed based on the ESHRE consortium recommendations (De Rycke *et al.* 2022). The discordance of the majority of the partially discordant cases (5/6) concerned a segmental aberration. This aligns with the findings of Victor *et al.* 2019 who reported that segmental aneuploidies exhibited a significantly different rate of clinical TE-ICM discordance compared with whole chromosome aneuploidies (Schoolcraft *et al.* 1999, Victor *et al.* 2019); however, technical artefacts cannot be excluded. Additional studies on this should be performed. In addition, all partially discordant cases detected in our study are also aneuploid for at least one chromosome and therefore unsuitable for transfer; consequently, the partial discordance observed here would most probably have no effect on the outcome of a routine embryo assessment. It is important to point out that in routine IVF, clinical decisions are ultimately based on the mosaicism level reported in PGT-A from a single TE biopsy, while the mosaic aberrations described above were identified by testing many more cells, as represented by the entire ICM or TE. The difference in sampling strategy implies that the mosaicism levels reported in PGT-A may either underrepresent or overrepresent the overall mosaicism of the embryo, depending on the biopsy site. However, no strong indications of this have been reported, with Chuang *et al.* showing that chromosomal consistency is not influenced by the biopsy site within the TE, whether proximal or distal to the ICM (Chuang *et al.* 2018).

To address the issue of TE sampling, previous PGT-A results of fourteen of the tested embryos were reviewed, showing a uniform concordance with the ICM and TE profiles. This indicates that a single trophectoderm biopsy, typically used in PGT-A, provides a representative chromosomal assessment for the entire embryo, further supporting its clinical utility. The random distribution of cells with differing chromosomal components within the TE is supported by previous studies, where biopsy sites in the TE, whether near to or far from the ICM, showed similar chromosomal profiles. The close alignment of genetic information within the TE, as well as the high concordance between TE and ICM, highlights the reliability of PGT-A in predicting the overall chromosomal status of the embryo.

Furthermore, given the distinct biological roles of the ICM and the TE in early human development, we aimed to investigate whether differences in the mtDNA copy number between these two cell populations could provide additional insights into embryo viability and implantation potential. The ICM gives rise to the fetus, while the TE contributes primarily to placental development and is a key driver of implantation. By quantifying mtDNA content separately in ICM and TE cells, we sought to explore whether mtDNA levels could serve as a biomarker not only for genomic integrity, as assessed through PGT-A, but also for implantation competence, which is largely mediated by the TE.

**Figure 3:**
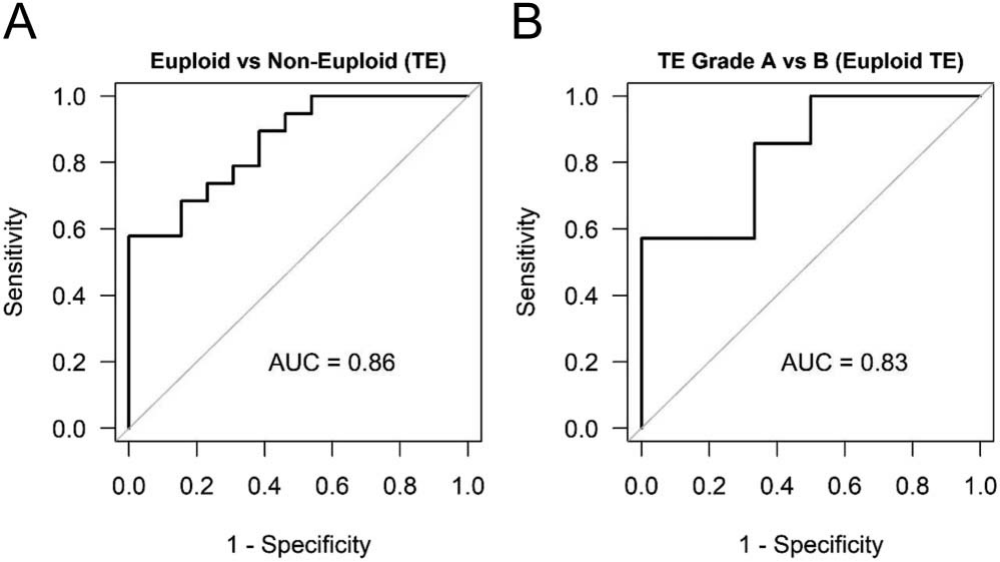
Graphical presentation of the ROC/AUC analyses between euploid and non-euploid results of the TE group (A) and between grade A and grade B of the trophectoderm in the euploid TE group (B).

To our knowledge, this study is the first to compare mtDNA levels between the entire cell population of the TE and the ICM of the same human embryo. A higher mtDNA copy number was observed in TE compared with the ICM of the same embryo. This can be explained by the difference in the timing of mtDNA replication, which occurs first in the TE to provide the energy needed for implantation (St John *et al.* 2010). ICM cells tend to retain their pluripotency for longer, which requires lower levels of energy production from the mitochondria (Fragouli *et al.* 2008). Furthermore, a higher mtDNA copy number was observed in the non-euploid ICM and TE compared with the euploid ICM and TE, respectively. This allows us to consider the mtDNA copy number as a complementary biomarker to ploidy status (Fragouli *et al.* 2015) alongside TE biopsy-based PGT-A, potentially adding to the genetic evaluation of embryos. Efforts are underway to establish an mtDNA quantity threshold for embryo assessment (Ravichandran *et al.* 2017) to facilitate prioritisation of embryos for transfer, most relevant when multiple euploid embryos are available. In our case, despite the limitation of a small sample size, we attempted to establish a potential mtDNA copy number threshold, which permits further validation in larger cohorts. In the TE sample group, the mtDNA copy number demonstrated a strong discriminatory ability between euploid and non-euploid results (area under the curve (AUC) = 0.86) (Fig. 3A) with a copy number threshold of 369 copies.

Finally, no significant association was observed between the embryo ploidy status and embryo morphological grading. This lack of relation may be attributed to the limited variability in the embryo morphology (average to excellent quality) within our cohort, which included embryos that met established morphological standards and were cryopreserved for prospective clinical application. Despite that, a significantly higher number of mtDNA copies were detected in the TE with an A-score than in the ones with a B-score. To our knowledge, this is the first report regarding the study of solely the TE morphology and the mtDNA copy number. Many scientific articles have studied the mtDNA copy number in regard to the morphology of the whole blastocysts and/or the chromosome profile via the PGT-A biopsy (Fragouli *et al.* 2017). Although the interpretation of this may be complex, an A-grade TE typically reflects a higher cellular density, hence a higher proliferative activity compared with a B-grade TE, which is characterised by a lower number of cells. This distinction suggests that embryos with TE with an increased cell number forming a cohesive epithelium at day-5 embryos may possess enhanced developmental competence, potentially translating to improved implantation. This could be highly informative as the PGT-A biopsy is obtained from the TE, and the mtDNA could also be evaluated as a combined biomarker, especially in cases where more than one PGT-A euploid and of good morphology embryos are available, and an additional marker evaluation will be supportive for a better assessment. We ended up with an mtDNA copy number threshold of 182, within the euploid TE sample group, where the mtDNA copy number showed a good discriminatory ability between the A-grade and B-grade morphology of the TE (AUC = 0.83) (Fig. 3B). Collectively, these observations support the potential role of the mtDNA copy number in refining embryo selection strategies.

Larger studies with more diverse sample sets are required to confirm the above associations and enhance their value, also addressing any technical limitations and variability that could have affected mosaicism detection sensitivity and data interpretation (Popovic *et al.* 2024). Even though, at the time of sample selection, no information regarding the pregnancy outcome of the couple was known or considered, a potential source of bias may be present since these could have been derived from couples with a successful IVF outcome.

Another limitation to the reproducibility of this study could be possible cross-contamination between TE and ICM; however, this risk was minimised by the preventive technical measures applied during TE and ICM dissection, as described in the materials and methods section. Furthermore, the vast majority of the analysed embryos (30 of 32) showed full, non-mosaic chromosomal findings or mosaic findings that were detected only in TE or only in ICM, providing evidence against cross-contamination.

Most importantly, studies assessing pregnancy outcomes are essential to evaluate the clinical relevance and implications of these findings (Viotti *et al.* 2023). As embryo selection continues to rely on a combination of morphological, genetic and emerging markers, these studies can contribute to the foundation for developing more specific, reliable and predictive approaches. More recent studies have increasingly utilised artificial intelligence to enhance these approaches (Coticchio *et al.* 2024, Bori *et al.* 2025), including its application in non-invasive prediction models (Del Arco de la Paz *et al.* 2024, Shan *et al.* 2024, Horta *et al.* 2025). Enhancing the precision of preimplantation evaluation ultimately holds the potential to improve clinical outcomes in assisted reproductive technologies.

In conclusion, and despite the small sample size that requires cautious interpretation, our study has addressed the hypothesis of discordance between the ICM and the TE of day-5 embryos, showing a high and significant concordance of their ploidy status. Thus, the produced data support the clinical validity of PGT-A and contribute to addressing concerns about PGT-A reliability in predicting an embryo’s true chromosomal status. Furthermore, the mtDNA copy number could be considered an additional biomarker for embryo viability, especially among euploid embryos. Our preliminary assessment indicates that the mtDNA copy number of the TE is higher than that of the ICM, which is consistent with the processes taking place during embryo development. The TE mtDNA copy number also appears to correlate with the morphology grade of the TE alone. More studies with larger clinical outcome results are needed to further address the above and to elucidate the relationship between mtDNA levels in both cell populations of the blastocyst.

## Declaration of interest

The other authors declare no conflict of interest. The study was approved by the Cyprus National Bioethics Committee (Protocol No. EEBK/EΠ/2024/30). Consents were obtained from all participating couples.

## Funding

The lead author’s research was funded by an internal Research Fund of The Cyprus Institute of Neurology and Genetics. The Open Access fee for this publication is covered by the Cyprus Institute of Neurology and Genetics.

## Data availability

All data supporting the findings of this study are presented within the manuscript. The raw sequencing datasets are stored in the department’s archive and are available from the corresponding author upon request, subject to institutional approval and applicable data-sharing regulations.

## Author contribution statement

The principal author contributed to all aspects of the research, including sample requirement, laboratory work, data processing and write-up. All authors approved the final version of the manuscript and agreed to be accountable for their contribution in the work.
